# orsum: a Python package for filtering and comparing enrichment analyses using a simple principle

**DOI:** 10.1186/s12859-022-04828-2

**Published:** 2022-07-23

**Authors:** Ozan Ozisik, Morgane Térézol, Anaïs Baudot

**Affiliations:** 1 Aix Marseille University, Inserm, MMG, Marseille, France; 2 Barcelona Supercomputing Center (BSC), Barcelona, Spain; 3 CNRS, Marseille, France

**Keywords:** Over-representation analysis, Enrichment analysis, Filtering, Neurodegenerative diseases

## Abstract

**Background:**

Enrichment analyses are widely applied to investigate lists of genes of interest. However, such analyses often result in long lists of annotation terms with high redundancy, making the interpretation and reporting difficult. Long annotation lists and redundancy also complicate the comparison of results obtained from different enrichment analyses. An approach to overcome these issues is using down-sized annotation collections composed of non-redundant terms. However, down-sized collections are generic and the level of detail may not fit the user’s study. Other available approaches include clustering and filtering tools, which are based on similarity measures and thresholds that can be complicated to comprehend and set.

**Result:**

We propose orsum, a Python package to filter enrichment results. orsum can filter multiple enrichment results collectively and highlight common and specific annotation terms. Filtering in orsum is based on a simple principle: a term is discarded if there is a more significant term that annotates at least the same genes; the remaining more significant term becomes the representative term for the discarded term. This principle ensures that the main biological information is preserved in the filtered results while reducing redundancy. In addition, as the representative terms are selected from the original enrichment results, orsum outputs filtered terms tailored to the study. As a use case, we applied orsum to the enrichment analyses of four lists of genes, each associated with a neurodegenerative disease.

**Conclusion:**

orsum provides a comprehensible and effective way of filtering and comparing enrichment results. It is available at https://anaconda.org/bioconda/orsum.

## Background

Enrichment analyses are widely used to investigate lists of genes of interest, such as genes differentially expressed or genes associated with disease variants. However, the outputs of enrichment analyses are often long lists of redundant annotation terms. These long lists make the interpretation, reporting and comparison of enrichment analysis results complicated.

Redundancy in enrichment results is often due to the hierarchical structure of certain annotation databases, such as Gene Ontology (GO) [1, 2] or Reactome [3]. In these databases, the annotation terms are linked by parent-child relationship where ancestor terms correspond to more general terms and descendant terms correspond to more specific terms. Importantly, ancestor terms annotate all the genes annotated by their descendants. The overlap of annotated genes causes the risk of both parent and child term to be significant in the enrichment analyses.

A first solution to overcome redundancy consists in using down-sized, non-redundant collections of annotation terms. Such collections include for instance “GO subsets”, also known as “GO slims”. GO subsets contain only a portion of the GO terms, providing a coarse-grained ontology. Generic and organism-specific GO subsets are maintained by the GO consortium and different communities [4, 5]. The hallmark collection of The Molecular Signatures Database (MSigDB) [6] is another down-sized collection of annotation terms. It contains 50 non-redundant terms obtained by clustering and filtering over 4000 terms from different databases. The enrichment analysis tool FunMappOne [7] has its own down-sized versions of the Kyoto Encyclopedia of Genes and Genomes (KEGG) [8], GO and Reactome databases. The annotation terms are stored in three hierarchical levels, ranging from the full list of terms to the summary list.

Down-sized annotation collections are built a priori; they hence allow directly obtaining non-redundant enrichment results. In addition, using a lower number of annotation terms implies less statistical tests. The multiple testing correction will hence be more gentle. The main drawback of down-sized annotation collections is that the terms may be too general or too specific for the user’s study. Additionally, these collections are not available in all the enrichment analysis tools. Finally, the user is dependent on the curators of these collections for updates; for instance, some GO subsets may become outdated while GO is updated.

The second solution to overcome redundancy in enrichment results is the clustering of the enriched annotation terms. The clustering methods calculate the similarity between two annotation terms either with statistical measures on the corresponding sets of annotated genes or with semantic similarity measures. Various semantic similarity measures exist. The most common ones depend on the frequencies of the assessed terms and of their closest common ancestor term in the annotation database [9, 10]. The reader is referred to [10] for a review on semantic similarity. RedundancyMiner [11] is a tool that clusters enrichment results. The similarity between two terms is measured by Fisher’s exact test on a contingency table storing the number of common and different genes between the two terms. G-SESAME [12] is an online set of tools associating the annotation terms based on semantic similarities. DAVID [13], ClueGO [14], clusterProfiler [15, 16], pathfindR [17] and ViSEAGO [18] are among the enrichment tools that provide additional functions to cluster enriched terms. While clusterProfiler and ViSEAGO use semantic similarity measures, the others use kappa statistics on annotated gene sets for clustering.

The third solution to overcome redundancy in enrichment analysis results is filtering. One widely used and highly cited tool in this category is REVIGO [19]. REVIGO is available as a web tool. It selects pairs of terms that are more similar than a threshold based on semantic similarity measures and compares them. In the comparison, REVIGO checks step by step whether one of the terms is very general, or less significant than the other, or a child term of the other. It then discards the term that satisfies the condition. The Cytoscape [20] app of STRING database [21], stringApp [22], provides functionality for enrichment analysis and removal of redundant terms. From two terms with high number of overlapping genes, the less significant one is discarded. A popular enrichment tool, g:Profiler [23, 24], also provided filtering options that considered term significance and parent-child relations on its web interface (before version e94_eg41_p11_5fca2e9) and in its previous R package, gProfileR. GOsummaries [25], which is an R package for visualizing enrichment results, uses gProfileR package for enrichment analysis and filtering.

Clustering and filtering approaches process the user’s enrichment results. They hence provide summarized results tailored to the input enrichment analyses. The tools developed for clustering and filtering can work with any enrichment analysis result; the user is thus free to choose any enrichment analysis tool. However, available clustering and filtering approaches are based on similarity measures that can be complicated to handle. It can indeed be complex for the users to set thresholds for similarity and anticipate the consequences on the obtained summarized results. Finally, many popular tools work with built-in annotation data. In this case, the user is dependent on the developers for updates.

Redundancy complicates the interpretation and reporting of enrichment results. Another challenge is comparing multiple enrichment analyses. Such comparisons are carried out, for example, when multiple lists of genes associated with different conditions are analyzed, or when the same list of genes is analyzed using different enrichment analysis tools. This challenge is particularly tedious because similar annotation terms can be as relevant as the exact term matches. BACA [26] is a tool for enrichment analyses of multiple gene lists. For each input gene list, BACA obtains enrichments through the DAVID web service. It then creates a bubble chart that presents the gene lists enriched in each term. BubbleGUM [27] is a tool that runs Gene Set Enrichment Analysis (GSEA) [28] on expression data by processing multiple conditions in pairs. Similar to BACA, the results are presented on a bubble chart allowing comparison of different analyses. FunMappOne and clusterProfiler, mentioned previously, can also work with multiple input gene lists; they produce plots that allow comparison of the enrichment results. ClueGO and pathfindR merge multiple results showing whether the term is common or specific to one of the results. However, to the best of our knowledge, no tool performs collective summarization considering multiple results together.

In this study, we present *orsum* (which stands for “over-representation summary”), a tool to filter enrichment analysis results. orsum has three main features: (i) The filtering process is straightforward, (ii) The ranking of the terms are considered in order to preserve the main biological information, (iii) Multiple enrichment results are filtered collectively.

## Implementation

orsum is a Python-based enrichment results filtering tool. The filtering is based on a simple principle: a term is discarded if there is a more significant term that annotates at least the same genes. In this case, the remaining more significant term becomes the representative term for the discarded term.

The inputs for orsum are enrichment analysis results containing term IDs ordered by statistical significance (from the most to the least significant; significance values are not given) and Gene Matrix Transposed (GMT) file. GMT is a common format to store annotation data, where each row stores an annotation term and the annotated genes.

orsum has two parameters related to the sizes of the considered terms. The first parameter, “minimum term size”, is used to discard terms annotating only a small number of genes. The default value is 10. The second parameter, “maximum representative term size”, is a threshold that limits the size of the terms that can represent other terms; it allows retaining terms annotating smaller number of genes in the filtered results. By default, this second parameter is not applied.

orsum starts by reading the input enrichment results and the GMT file. Then, an initial representative term list is created. At this point, this list contains the full list of terms present in the input enrichment analysis results. The terms are accompanied by their ranking values corresponding to the order they appear in the enrichment results. In the case of multiple input enrichment results, the input term lists are merged, duplicate terms are removed, and these terms get the best rank they have in any of the input enrichment results.

Next, the filtering process begins. Starting with the top-ranked terms, pairs of terms are checked iteratively. If a better ranked term covers all the genes annotated by a lower ranked term, then the lower ranked term is discarded and represented by the better ranked term. In other words, more significant ancestor terms represent their less significant descendant terms.

orsum outputs multiple files providing both simplified and detailed views of the filtered results:

- An HTML file with the filtered list of representative terms. The user can click on each representative term to see the discarded terms.
- Two TSV files (“-Summary.tsv”, “-Detailed.tsv”) with the information contained in the HTML file in different formats. “-Summary.tsv” file contains only representative terms while “-Detailed.tsv” additionally contains the terms represented by them.
- A heatmap presenting the top representative terms, colored according to the quartile of their ranks in each input enrichment result.
- A barplot presenting the top representative terms and how many terms they represent.

In the output figures, the number of terms to be presented is adjustable (default and maximum 50).

orsum is implemented in Python 3. The source code is available on https://github.com/ozanozisik/orsum and the package can be installed via bioconda (https://anaconda.org/bioconda/orsum).

## Results

### Use case: Four neurodegenerative diseases

orsum is designed to filter enrichment analyses and compare results from different studies. In order to illustrate orsum, we applied it to the enrichment analysis results of four gene lists, each associated with a neurodegenerative disease. These diseases are Alzheimer’s disease (AD), amyotrophic lateral sclerosis (ALS), Huntington’s disease (HD) and Parkinson’s disease (PD). As these diseases are all neurodegenerative diseases, in addition to the filtering of enrichment results, the common and different enriched annotation terms between diseases are also of interest.

We obtained gene lists associated with the four diseases from DisGeNET [29, 30] (on 20.12.2021). The queried disease terms are:

- C0002395 Alzheimer’s Disease
- C0002736 Amyotrophic Lateral Sclerosis
- C0020179 Huntington Disease
- C0030567 Parkinson Disease

**Fig. 1:**
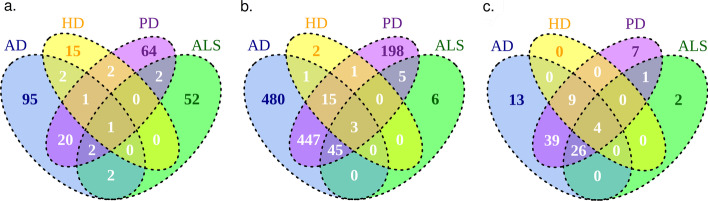
Venn diagrams presenting **a** the overlap of genes associated with each disease, **b** the overlap of GO Biological Process terms enriched in genes associated with each disease, **c** the overlap of GO Biological Process terms enriched in genes associated with each disease after collective filtering by orsum

We selected the genes with gene-disease association score greater than or equal to 0.3. The number of selected genes are 123 for AD, 59 for ALS, 21 for HD and 92 for PD. These disease-associated genes display some overlap (Fig. 1a). We performed enrichment analyses using GO Biological Process annotation terms with g:Profiler (version e104_eg51_p15_3922dba). We ran g:Profiler with the default options and obtained statistically significant terms. AD genes are enriched in 991 GO terms, ALS genes in 59 GO terms, HD genes in 22 GO terms, and PD genes in 714 GO terms. These long lists of terms that are obtained for all diseases but HD illustrate the difficulty in interpreting enrichment results. The enriched terms have high overlaps but there are also many terms unique to each neurodegenerative disease (Fig. 1b).

We applied orsum to the four enrichment results collectively. We used the default settings except for the number of terms to be plotted; minimum term size was 10, maximum representative size threshold was not used, and top 20 terms were plotted. The command is given below:
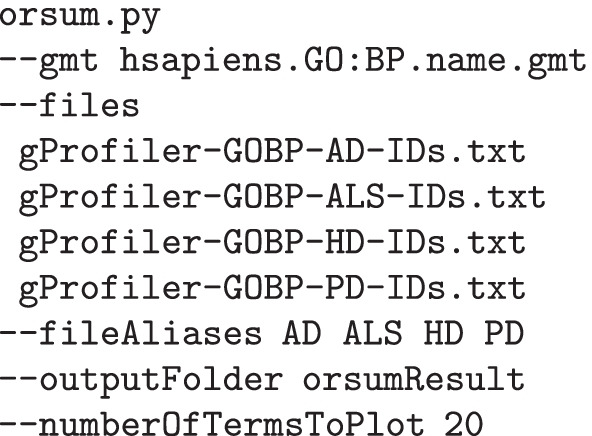


The four enrichment results collectively lead to 1203 enriched terms. From these, 40 terms were discarded because they annotated less than 10 genes. And finally, with the application of orsum filtering, the number of terms decreased from 1163 to 101.

In Fig. 1b we saw that enriched terms have high overlap but also there are many terms unique to each disease. After summarization, this distribution is conserved (Fig. 1c). However, common and specific terms can be analyzed more easily.

**Fig. 2:**
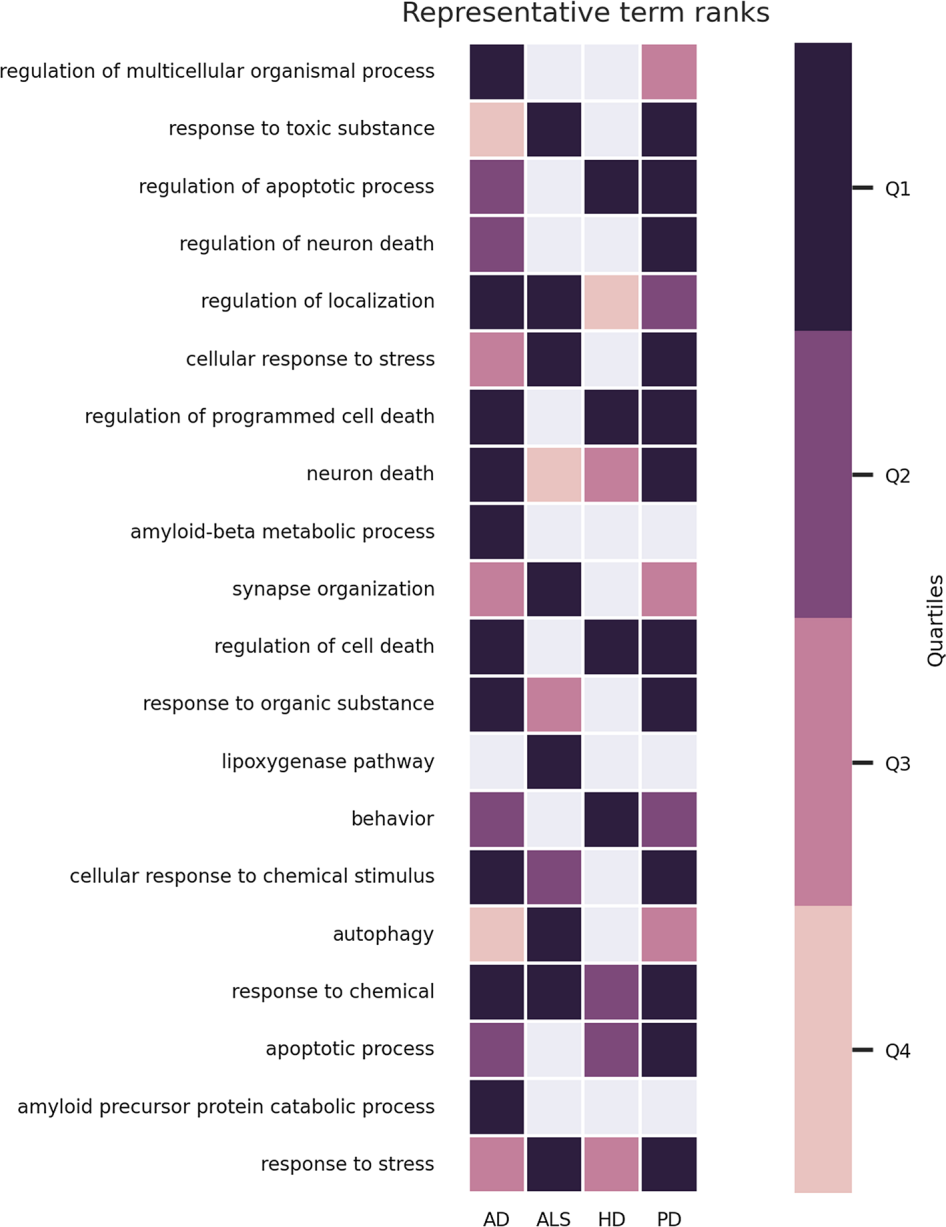
Top 20 representative terms and the quartiles their ranks belong to according to each input enrichment result

In Fig. 2, the top representative terms and the quartiles of their ranks according to each input enrichment result are presented. In this figure we can easily see the most significant representative terms and analyze whether these terms are shared and similarly important among the different diseases. For example, we see that “regulation of localization”, “neuron death”, “response to chemical” and “response to stress” are significant for all the analyzed neurodegenerative diseases. However, “regulation of cell death” and associated terms are significant for AD, HD and PD but not for ALS. In the figure, we can also identify the terms that are specific to a given disease. For example, “Amyloid-beta metabolic process” and “amyloid precursor protein catabolic process” are the two terms specific to AD among top 20 representative terms. Extracellular plaque deposits of the amyloid-$\beta$ is one of the hallmark pathologies of AD [31]. Another example is “Lipoxygenase pathway”, a term specific to ALS, which is significant due to paraoxonase genes associated with the disease [32].

**Fig. 3:**
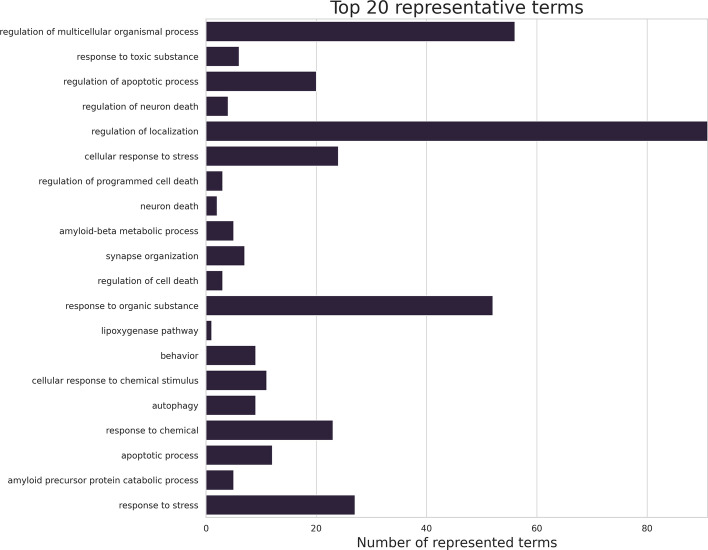
Top 20 representative terms and the number of terms they represent

Among the top representative terms, “regulation of multicellular organismal process”, “regulation of localization” and “response to organic substance” represent more than 50 discarded terms (Fig. 3). These terms are general terms each annotating more than 2500 genes. We observed that there is a moderate correlation between the number of genes a term annotates and the number of terms it represents (Spearman’s rank correlation, r=0.56, *p*=1.22E-09).

The obtained results show that orsum can filter long lists of enrichment results and highlight common and specific terms in the different enrichment results.

### Comparison with REVIGO

As stated in the introduction, REVIGO [19] is a widely used approach to filter enrichment analysis results. We devised two strategies to compare orsum and REVIGO based on the number of representative terms obtained after filtering.

In the first strategy, we applied orsum and REVIGO to the enrichment analysis result of each of the four neurodegenerative diseases that we explored as a use case. REVIGO was run with default parameters except that we selected Homo sapiens as the species; the inputs were the GO terms with their respective *p*-values. For the sake of comparison with REVIGO, please note that here, orsum needs to be applied separately for each disease and not using its collective filtering feature. In this comparison we observed that REVIGO performed limited filtering (Table 1). Among these results we can comment on the results for HD. In this case, both orsum and REVIGO performed filtering on terms related to cell death, in different ways; as it is the way orsum works, ancestor terms that are more significant than their descendants were never eliminated in favor of the descendants in orsum, while REVIGO favored descendant, more specific terms even if they are less significant. orsum performed additional filterings by making “behavior” representative for “learning or memory” and “memory”, and “response to chemical” representative for “response to oxygen-containing compound”.

**Table 1:** Numbers of annotation terms in the enrichment analysis results of four neurodegenerative disease gene lists, in total and after filtering by orsum and REVIGO

Disease	Total number of terms	Number of terms after orsum filtering	Number of terms after REVIGO filtering
AD	991	79	540
ALS	59	34	51
HD	22	12	17
PD	714	104	415

**Fig. 4:**
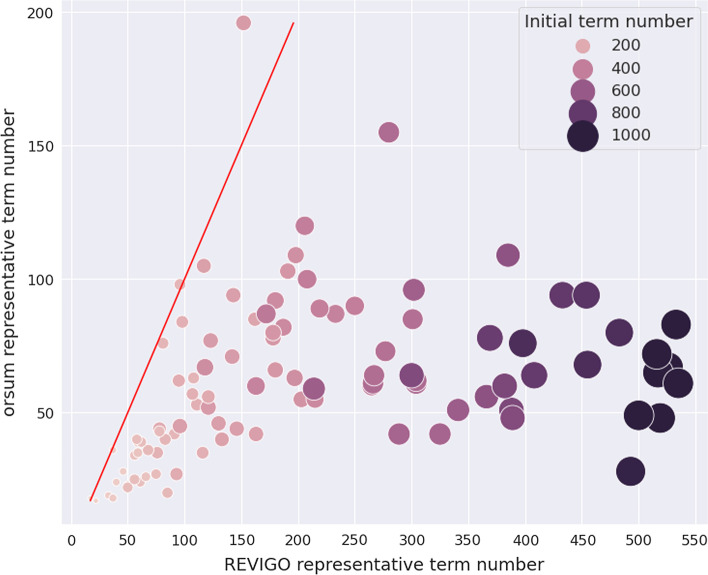
Numbers of representative terms resulting from orsum and REVIGO applied to the enrichment analyses of artificially generated gene lists. Each point corresponds to an enrichment analysis result obtained for one of the 100 artificially generated gene lists. The size and color of a point indicates the number of terms in the original enrichment analysis result. The red line shows the coordinates where the representative term numbers in orsum and REVIGO are equal

In the second strategy, we devised a systematic approach using enrichment analysis results of artificially generated gene lists. We first generated 100 artificial query gene lists to perform enrichment analysis. However a random gene list will rarely be significantly enriched in any annotation terms. Therefore, we built each query gene list using both random genes and genes annotated by a given annotation term. From GO Biological Process terms, we randomly selected a term that annotates more than 100 genes. Let’s say the term annotates *n* genes. We randomly selected *n*/2 genes annotated by the term, and we added *n*/2 other random genes, getting an artificial query gene list of size *n*. Next, we performed enrichment analysis on GO Biological Process terms using the hypergeometric test. We selected the terms with *p*-value$<0.05$ after Bonferroni correction. Finally, we submitted the enrichment results to orsum and REVIGO and counted the representative term numbers obtained in the filtered results. For REVIGO, we benefited from its REST API. REVIGO allows queries up to 2000 terms but we encountered errors, so we limited our enriched terms list to the top 1000. This limitation concerned only 7 out of the 100 iterations. We observed that orsum resulted in a smaller filtered terms list in 97 out of 100 iterations (Fig. 4).

## Discussion

We present orsum, a tool to filter enrichment results. We applied orsum on the enrichment results of four neurodegenerative diseases as a use case. We also compared orsum with the widely used REVIGO approach.

The core of orsum is its filtering principle: more significant ancestor terms represent their less significant descendant terms. In the use case, we demonstrated that this effectively reduces the number of the enriched terms to a more interpretable level. The main advantage of this principle is its simplicity. The users can easily conceive how orsum filters the terms. The output of orsum is foreseeable and, looking at the representative terms, the user knows that the most significant terms are still belonging to the output list and representing their less significant descendant terms. This simple filtering principle has three consequences: (i) There is a moderate correlation between the number of genes a term annotates and the number of terms it represents. As expected, larger terms represent more terms. (ii) When a term is more significant than its ancestor, both terms are kept as representative terms. Although the redundancy is retained in such a case, this ensures that more significant and specific biological information is presented to the user. (iii) orsum works with only the annotation databases that are hierarchically organized such that terms are in a parent-child relationship, e.g. GO and Reactome. For the summarization of enrichment analysis results obtained from databases that are not hierarchically structured, we recommend the clustering approaches.

orsum can process multiple lists of enriched terms resulting from the analysis of different conditions or from the analysis of the same condition with different tools. This allows comparing and integrating different enrichment results. It should be noted that when applying different tools on the same input data, a proper multiple testing correction might be required.

As stated in [19], it is difficult to make a quantitative comparison between enrichment result summarization tools as their success is based on subjective measures like interpretability of the final terms list. We should stress that these tools are aimed to ease the exploration of enrichment results and the user will choose the one that fits to their need and perspective. However, for the sake of completeness, we compared the number of representative terms obtained after applying orsum and REVIGO. We observed that orsum outperformed REVIGO.

Methods that use semantic similarity are critically dependent on the similarity threshold parameters adjusted by the user. For example, in REVIGO, pairs of terms that are more similar than the user adjusted similarity threshold are compared. Additionally there are parameters that cannot be tuned by the user in the REVIGO algorithm. orsum algorithm is not dependent on any parameters; it checks the ranks of the terms and it checks whether all the genes annotated by one term are annotated by the other. The two parameters of orsum are not mandatory, they can improve the final results based on the user’s requirements. The minimum term size parameter is used to remove the terms that annotate very small numbers of genes; many other terms might annotate the same set of genes. The maximum representative term size parameter is useful when a term that is more general than the user’s requirement is very significant and therefore represents many other terms. Our perspective here is that, if a general term is statistically significant, then this should be highlighted and presented to the user as representative for its less significant descendant terms. The user can further examine the detailed results to check for discarded terms. The user might also choose to see the smaller, descendant terms in the orsum output. Setting the maximum term size parameter to a low value prevents a large term to be representative of other terms. More specific terms remain in the filtered results. Both parameters of orsum are straightforward and their results are foreseeable.

orsum shares the advantages of filtering tools. It runs on enrichment analysis results provided by the user, which means that the user is free to choose any enrichment analysis tool. In addition, as the resulting terms are selected from the input, they are study-specific. In contrary to existing tools, the annotation data (GMT file) is provided by the user. This makes it possible to use the same annotations as the ones used in the enrichment analysis. The user also does not need the developers to update the files in a need for using up-to-date information.

## Conclusions

In this study, we developed orsum, a Python-based tool for the filtering of enrichment analysis results. orsum is easy to use, the applied principle for filtering is easy to understand, the sizes of the resulting representative terms can be adjusted, and the output files are informative even at a quick glance. orsum’s ability to work with multiple enrichment results will allow it to be used in comparative or integrative studies, for instance, in the investigation of multiple diseases or multiple -omics datasets.

## Availability and requirements

Project name: orsum (v1.4)

Project home page: https://anaconda.org/bioconda/orsum

Operating system(s): Platform independent

Programming language: Python

Other requirements: Python 3.6 or higher

License: MIT License

Any restrictions to use by non-academics: None

## Data Availability

The source code of orsum is available on https://github.com/ozanozisik/orsum. orsum can be installed via bioconda (https://anaconda.org/bioconda/orsum). All data and materials related to the use case and the comparison with REVIGO are available on https://doi.org/10.5281/zenodo.6036031.

## Abbreviations

AD: Alzheimer’s disease
ALS: Amyotrophic lateral sclerosis
GMT: Gene matrix transposed
GO: Gene ontology
GSEA: Gene set enrichment analysis
HD: Huntington’s disease
KEGG: Kyoto encyclopedia of genes and genomes
MSigDB: The molecular signatures database
PD: Parkinson’s disease
